# Latest advances on the role of P2X Receptors in colorectal inflammation and cancer

**DOI:** 10.1007/s11302-026-10146-6

**Published:** 2026-03-18

**Authors:** Luigia Ruo, Federica Fortuna, Marianna Grignolo, Anna Pegoraro, Elena Adinolfi

**Affiliations:** Department of Medical Sciences, Section of Experimental Medicine, University of Ferrara, Ferrara, Italy

**Keywords:** P2X receptors, eATP, Colon, IBD, Colon carcinoma

## Abstract

Extracellular adenosine triphosphate (eATP) is a hallmark of inflammatory and tumor-associated microenvironments, where it functions as a key extracellular signalling molecule through activation of purinergic receptors. In the gastrointestinal tract, and particularly in the colon, eATP-mediated signalling regulates epithelial barrier function, neuroimmune interactions, and immune responses. P2X receptors, a family of eATP-gated ion channels, are differentially expressed across epithelial, neuronal, and immune cell populations and are increasingly recognized as contributors to colonic pathophysiology. This review summarizes current evidence on the roles of P2X receptors in inflammatory bowel disease and colon carcinoma, highlighting their involvement in intestinal inflammation, visceral hypersensitivity, immune cell activation, and tumor-associated processes. We discuss how dysregulated P2X receptor signalling contributes to chronic inflammation and supports a tumor-promoting microenvironment, while also emphasizing the context- and cell-type-specific nature of these responses. Finally, we outline emerging therapeutic strategies targeting P2X receptors and underscore the importance of personalized approaches based on receptor expression patterns within the colonic tissue.

## Introduction

**Fig. 1:**
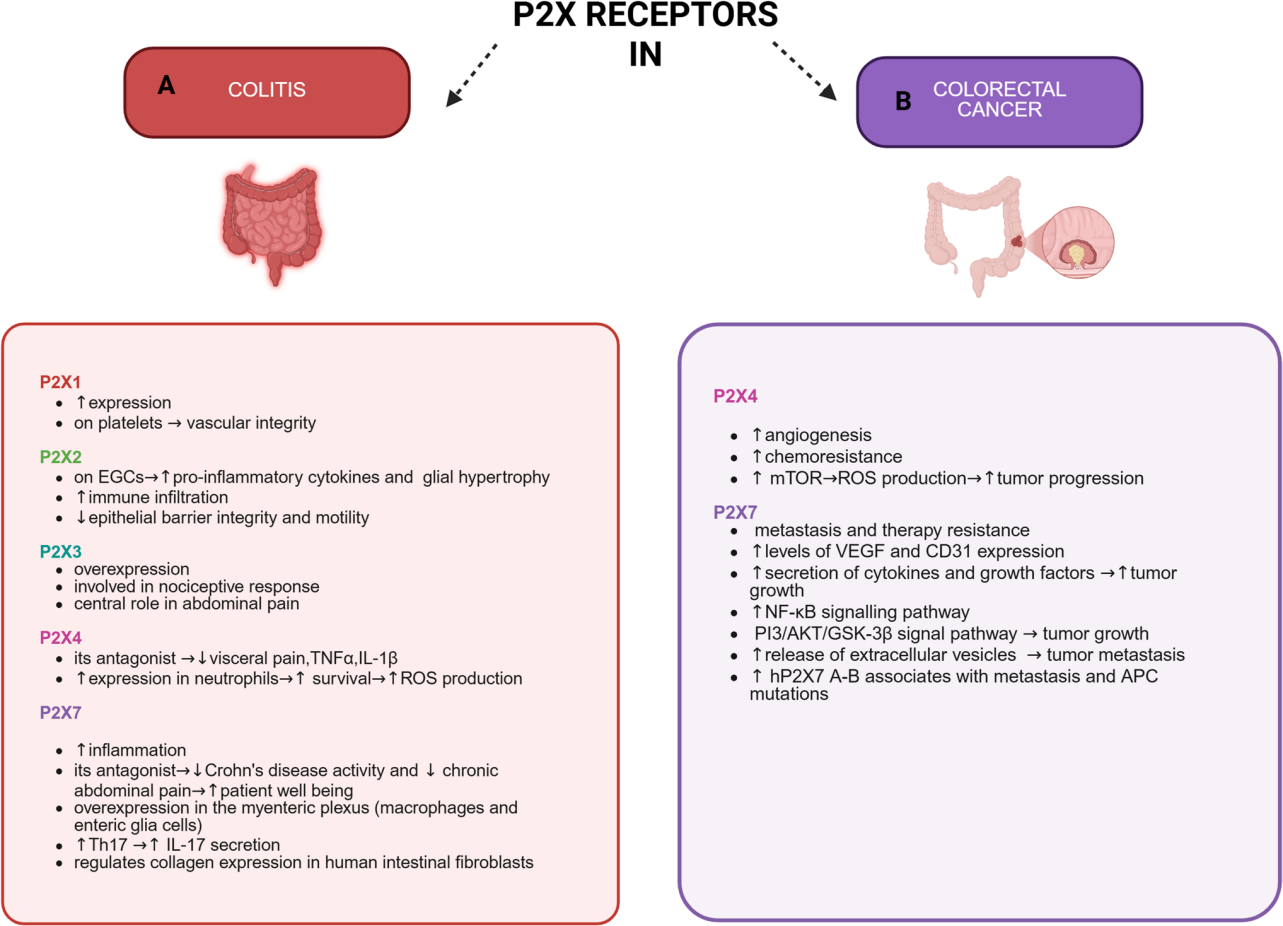
Schematic illustration of the role of P2X receptors in colitis and colorectal cancer. **A** In colitis, P2X1, P2X2, P2X3, P2X4, and P2X7 receptors contribute to pain, inflammation, immune cell infiltration, and tissue damage. **B** In colon cancer, P2X4 and P2X7 receptor activation promotes cell survival and growth, angiogenesis, EVs release, metastasis and therapy resistance

Extrcellular ATP (eATP) is a major component of inflammatory and tumoral microenvironments, where it can reach high micromolar concentrations [1–7] and acts as an extracellular messenger, affecting immune responses, cytokine release, cell proliferation, migration, and differentiation [8–10]. Of interest, the gut was the anatomical site where purinergic signalling was first identified, and, over time, gastrointestinal disorders have been the focus of several researchers interested in developing purinergic signalling-targeting therapies [11]. ATP is released into the extracellular space both passively by necrotic cells and actively by pannexins, connexins, ABC cassette proteins, and extracellular vesicles [12, 13]. Once outside of the cell, ATP binds and activates P2X and P2Y receptors [14]. P2X receptors are the main sensors of eATP, whereas P2Y G-protein-coupled receptors generally bind other nucleotides [15]. P2Xs are a family of cation channels comprising seven subunits (1–7) that are involved in several physiopathological conditions. All subunits share a general structure consisting of two transmembrane and intracellular domains, plus a large extracellular region containing the ATP-binding sites [16, 17]. All P2X subunits, with the exception of P2X7 can assemble either as homo- or heterotrimers, giving rise to receptors with different properties [18]. Heterotrimers are particularly significant because they often combine the kinetic and pharmacological profiles of their parent subunits, creating unique signaling pathways in tissues such as sensory neurons and smooth muscle [19]. P2X ion channels differ in cell type expression and sensitivity to ATP. P2X1, P2X2, P2X3, P2X4, P2X5, and P2X6 receptors exhibit high affinity for ATP in the low micromolar range [18]. In contrast, P2X7 requires millimolar concentrations of the agonist to be activated [18, 20]. P2X1 and P2X3 show fast desensitization kinetics, while P2X2, P2X4, P2X5, and P2X7 are slowly desensitizing [20], with P2X7 also allowing the opening of a large, unselective macropore, which mediates the passage not only of small ions but also of molecules of higher molecular mass [21]. P2X5 and P2X6 are less studied as they are generally not functional as homotrimers [20, 22–25]. Here, we will provide an overview of the latest advances on the role of P2X receptors in colon inflammation and cancer (Fig. 1). In the gut, the P2X1 receptor is expressed in excitatory motor neurons and in sympathetically innervated smooth muscles [11, 26]. P2X2 and P2X3 are present in almost all neurons innervating the gastrointestinal system [11, 26]. The P2X4 receptor is expressed in the intestinal crypts, immune cells, and excitatory motor neurons [18, 26]. Possibly the most expressed colonic P2X receptor is P2X7, which is present on epithelial cells, glial cells, mast cells, macrophages, lymphocytes, and enteric ganglia [11, 26].

## P2X in colonic inflammation

Inflammatory bowel diseases (IBDs), including Crohn's and ulcerative colitis, are chronic inflammatory pathologies of the gastrointestinal tract due to genetic predisposition, environmental factors, such as diet and smoking [27], and gut dysbiosis [28]. Although Crohn's disease and ulcerative colitis exhibit distinct morphological and clinical features, they share a common genetic background, with at least 29 susceptibility loci identified as common to both conditions. Advanced IBDs can be treated with anti-TNF drugs, but the lack of response of some patients to this therapy prompts the search for alternative therapeutic approaches to IBDs [29]. Several animal preclinical models have been developed to mimic inflammatory colitis [30]. One approach involves orally administering irritants to animals (generally mice or rats). Dextran Sodium Sulphate (DSS), 2,4,6-Trinitrobenzene Sulphonic Acid (TNBS), and 2,4-Dinitrobenzene Sulfonic Acid (DNBS) are the most commonly used reagents in these models. An alternative approach to inducing colitis-like symptoms in animals is a surgical model called the post-operative ileus model [31]. In this case, following an abdominal surgery, one can analyze the effects of a transient inhibition of intestinal motility. Wang and colleagues have recently demonstrated that P2X1 expression is increased in patients with colitis and in DSS-treated mice. These authors also found that P2X1 null mice had a mucosal barrier protected from DSS-induced colitis, thanks to reduced neutrophil infiltration, diminished alterations in the microbiome, and reduced expression of genes related to inflammation. Moreover, P2X1 inhibition improved the efficacy of anti-TNF-α therapy. [32]. However, P2X1 null mice also exhibit increased intestinal bleeding associated with colitis [33]. Ablation of P2X1 leads to massive intestinal bleeding and regenerative anemia caused by both impaired platelet degranulation and the mobilization of hyperactivated neutrophils. P2X1-deficient neutrophils exhibit an altered, proinflammatory phenotype, driven in part by elevated G-CSF levels that directly contribute to intestinal blood loss and promote a paradoxical increase in extra-intestinal, fibrin-rich thrombosis [33]. The central role of P2X1 in neutrophils, leading to increased infiltration of these cells into tissues, is confirmed by studies associating the receptor's activity with reduced gastric cancer progression [34, 35]. These studies show that low expression of P2X1 on neutrophils favors immune escape by increasing PDL-1 levels on lymphocytes [34] and by promoting the growth, migration, and invasion of cancer cells [35].

Similarly to P2X1, P2X2 deletion or pharmacological blockade also affects colitis development in experimental models by reducing enteric gliosis [36]. Enteric gliosis is a feature of IBDs, characterized by structural and functional alterations in enteric glial cells (EGCs) that contribute to neuroimmune dysregulation in the gut [37]. Stimulation of the P2X2 receptor expressed on EGCs induces glial hypertrophy and increases production of proinflammatory cytokines [36]. In a postoperative ileus model that mimics a colitis-like condition, P2X2 activation promotes immune infiltration and impairs epithelial barrier integrity and motility. Pharmacological inhibition or genetic silencing of P2X2 reduces ATP-induced Ca^2^⁺ signalling in EGCs and gliosis, and alleviates inflammation in this model. These findings establish the role of P2X2 in glial activation and suggest the receptor as a therapeutic target for intestinal inflammatory diseases [36].

Since P2X3 is involved in nociceptive responses, it also plays a central role in abdominal pain [38–40]. Abdominal pain and visceral sensitivity are some of the most common symptoms of IBD, which are due to the release of inflammatory mediators that hit peripheral nerves. The P2X3 antagonist A-317491 reduces visceral hypersensitivity in acute colitis and post-inflammatory models [39]. Valdez-Morales and colleagues also demonstrated that dorsal root ganglion (DRG) neurons in mice with DNBS-induced colitis became overexcitable due to P2X3 overexpression [40]. Exposure of control DRG neurons to TNF-α induced a level of hyperexcitability comparable to that observed in neurons derived from DNBS-treated tissue. This TNF-α–evoked nociceptive response was prevented by the selective P2X3 receptor antagonist A-317491 [40].

Of interest, visceral hypersensitivity associated with DNBS-induced colitis could also be relieved by P2X4 blockade in rats [41]. Oral treatment with two different P2X4 selective antagonists (NC-2600, NP-1815-PX) proved more efficacious than dexamethasone in reducing not only visceral pain but also TNF-α and IL-1β levels. The same treatments also prevented the loss of occludin via an NLRP3 inflammasome-dependent pathway [41]. These data are in line with previous studies demonstrating a role for P2X4 and P2X7 receptors in regulating ion transport in rat colonic epithelia [42]. Ballout and colleagues demonstrated functional responses of both receptors in the serosa, while mucosal responses were mainly attributable to P2X7 [42]. Additional indirect evidence for the importance of the P2X4 receptor in the pathogenesis of colitis comes from studies conducted in mice lacking the ecto-ATPase ENTPD8 [43]. In these animals, the absence of ecto-ATPase activity leads to ATP accumulation, and following DSS treatment, colonic injury is markedly exacerbated. This phenotype is accompanied by increased P2X4 expression in neutrophils, leading to enhanced metabolism, prolonged survival, and increased production of reactive oxygen species [43].

Amongst the other members of the family, the P2X7 receptor is best known for mediating proinflammatory responses [7, 31]; therefore, it is not surprising that many studies have analysed the role of this receptor in colitis [11]. The P2X7 receptor was found to be upregulated in several murine models of IBD [44–48] and in Crohn's human colonic mucosa [48]. Based on these studies, a P2X7 inhibitor was evaluated in clinical trials in patients with Crohn's disease, where it reduced disease activity and improved patient well-being by alleviating chronic abdominal pain [49]. Nevertheless, P2X7 antagonism did not significantly reduce IBD-related inflammatory biomarkers, ultimately leading to discontinuation of the clinical program [49]. Subsequent preclinical studies further analysed the role of P2X7 in the pathophysiology of colitis. Some investigators identified novel P2X7-targeting therapeutic strategies based either on innovative small molecules [50] or nanobodies [51]. Notably, an adeno-associated viral (AAV) vector has been developed to achieve sustained in vivo delivery of a P2X7-blocking nanobody [51, 52]. In particular, AAV-mediated delivery of a P2X7-blocking nanobody reduces inflammation and disease severity in DSS-induced colitis by downregulating proinflammatory mediators [51]. Similar results were obtained by Figliuolo and colleagues, who analyzed colonic immune cell infiltration in *p2* × *7-/-* mice following colitis induction with two different chemical agents [53]. Genetic ablation of P2X7 led to increased Treg infiltration and IL-10 and TGFβ1 release, and decreased T-helper cell and macrophage infiltration in the colon mucosa [53]. The presence and function of P2X7 on neurons innervating the colon, particularly those of the myenteric plexus, have been controversial. Some studies, using antibodies that are not always specific for the receptor, have reported P2X7 expression in these neurons and have proposed a role for the receptor in either neuronal death [46] or, conversely, neuronal protection [54]. However, a recent study by Nicke and colleagues, using multiple complementary strategies to detect P2X7 expression, excluded the possibility that this receptor is expressed by myenteric plexus neurons [55]. Using a combined approach involving P2X7-EGFP transgenic mice, a nanobody targeting the receptor, and highly specific antibodies, these authors ruled out P2X7 expression in these neurons while confirming P2X2 and P2X4 expression [55]. The same authors reported overexpression of P2X7 in other myenteric plexus cells, including macrophages and enteric glia, suggesting that these cells are responsible for P2X7-dependent inflammatory phenotypes in colitis [55]. A recent study also attributed a role to the P2X7 receptor in the mechanism by which *Porphyromonas gingivalis* (*P. gingivalis*) exacerbates colonic inflammation [56]. *P. gingivalis*is a gram-negative bacterium generally associated with periodontitis that is also linked to IBD [57] and colon cancer [58]. In mice, *P. gingivalis* ingestion, associated with TNBS treatment, intensified colonic and hepatic damage and increased mucosal bacterial permeability. Of interest, this phenotype was at least partially P2X7-dependent, as demonstrated by experiments in null mice [56]. Furthermore, WT mice showed higher levels of Th17 cells, whereas P2X7-deficient mice showed an increase in Tregs [56]. These data are in line with other reports demonstrating that P2X7 receptors play an important role in IL-17 secretion and Th-17-mediated responses [59–63]. Notably, P2X7 has also been implicated in regulating collagen expression in human intestinal fibroblasts. Furthermore, P2X7 deficiency in mice results in increased collagen deposition and up-regulation of multiple profibrotic markers in experimental models of intestinal fibrosis [64].

## P2X in colon carcinoma

The high content of eATP, resulting from necrotic cancer cell death, active secretion, and immune responses, is an established characteristic of the tumor microenvironment [65] and has even been exploited to develop antitumoral agents released solely at cancer sites [66, 67]. Moreover, the levels of eATP increase following classical antitumor interventions such as chemo or radiotherapy [68, 69] and can, in turn, due to the activity of ectonucleotidases CD39 and CD73, cause a rise in the levels of adenosine, a major derivative of ATP hydrolysis, and a known immunosuppressive and vascularization-promoting agent in cancer [70]. Therefore, it is not surprising that P2X receptors have been implicated not only in colorectal phlogosis but also in colon carcinoma (CRC) [71], which is the third most commonly diagnosed cancer worldwide [72]. Risk factors for this tumor include diet, alcohol, a sedentary lifestyle, and chronic gut inflammatory conditions. [73]. The incidence of CRC in young adults is increasing, with causes poorly understood and requiring elucidation [74]. To date, studies associating P2X receptors with colon carcinoma have focused on P2X4 and P2X7, whereas a role for P2X1 and P2X3 was suggested mainly in gastric cancer [34, 35, 75].

Several studies have reported that the P2X7 receptor contributes to tumor growth, progression, and therapy resistance [8, 12, 13, 65]. It is therefore not surprising that the P2X7 receptor is associated with poor overall survival and metastasis in CRC and has been proposed as a prognostic marker for the disease [76–78]. Our group was the first to demonstrate that overexpression of P2X7 in CT26 colon carcinoma cells increased tumor growth and neovascularization, affecting VEGF secretion and CD31 expression [79]. Similar data were obtained by Yang and colleagues in an orthotopic model of CRC [80]. P2X7-overexpressing tumors also showed stemness features and exhibited augmented release of CCL2 and CCL5, leading to increased recruitment of tumor-associated macrophages. P2X7 overexpression in CRC cells activated NF-κB signalling, suggesting that this pathway may be involved in P2X7-dependent oncogenic transformation [80]. Another signalling axis responsible for P2X7-mediated tumor growth, glycogen accumulation, and angiogenesis is the PI3K/AKT/GSK-3β pathway [81]. This same axis was also implicated in P2X7-dependent growth of two human colon carcinoma cell lines, SW620 and HCT116, both in vitro and in vivo [82]. Treatment of both cell lines with P2X7 antagonist A438079 or the PI3K inhibitor LY294002 reduced their proliferation rate. However, combined antagonism did not show synergistic activity, thus supporting the notion that PI3K will be activated downstream of P2X7 [82]. The selective P2X7 antagonist A438079 was shown to reduce proliferation and migration in CRC cell lines HCT116 and SW620 [83]. The same drug significantly and dose-dependently reduced SW620 cell growth in a subcutaneous murine xenotransplantation model, promoting apoptosis by activating multiple caspases [83]. P2X7 antagonism has not consistently proven effective in reducing tumor growth in murine models of colon carcinoma. For example, in our hands, intraperitoneal administration of the inhibitor AZ10606120 at a dose of 2 mg/kg, delivered every three days, did not reduce tumor growth in xenografts derived from either HCT116 or CT26 cells in syngeneic subcutaneous models [62]. In contrast, administration of the same inhibitor in a syngeneic dissemination model proved effective, markedly reducing the formation of pulmonary metastases [62]. These discrepancies between experimental models may be attributable to the specific model employed and to the involvement of the immune system, which, in our case, proved central to the effects of P2X7 blockade [62]. In any case, we confirmed the overexpression of both functional human P2X7 splice variants (P2X7A and P2X7B) in metastatic forms of colon carcinoma and in patients carrying APC mutations and demonstrated a correlation with the expression of another key component of the purinergic signaling cascade in cancer, the A2A receptor. We also provided evidence that dual inhibition of P2X7 and A2A effectively reduces tumor growth in the subcutaneous CT26 tumor model. Notably, pharmacological blockade of both receptors also decreased circulating levels of IL-17 and IL-23 in both tumor-bearing and tumor-free control mice [62]. Finally, we demonstrated for the first time that extracellular vesicles (EVs) released by colon carcinoma cells following P2X7 activation are capable of promoting tumor metastasis in vivo, and that they lead to increased levels of circulating EVs in the blood of tumor-bearing mice 14 days after inoculation [62]. Moreover, we showed that P2X7 blockade effectively reduces the release of EVs [62, 84] and mitigates their in vivo effects [62]. This finding may be significant given the recognized role of EVs in promoting tumor metastasis [74] and the current lack of effective pharmacological agents to limit their release [85]. The role of EVs released following P2X7 activation may be important not only in cancer but also in neurotransmission and in cross-talk among neurons, astrocytes, and microglia, with additional implications for the study of colon pathologies [84, 86–88]. Interestingly, another P2X receptor, P2X4, mediates tumor-promoting effects dependent on EVs released by tumor cells. Palinski and colleagues recently demonstrated that human umbilical vein endothelial cells (HUVECs) exposed to EVs isolated from sarcoma patient biopsies overexpress P2X4. In this context, P2X4 is central to the formation of new vessels, and its blockade or silencing results in a significant reduction in blood vessel formation and branching, dependent on cancer's EVs exposure [89]. P2X4 was also associated with the progression of different tumors, including breast, prostate, hepatic, and colon carcinomas [90–96]. Possibly the most relevant recent work addressing the role of the P2X4 receptor in colon carcinoma is that published by Schmitt and colleagues, who demonstrated that this receptor is involved in the development of chemoresistance [95]. Specifically, in patient-derived CRC organoids, ATP released by dying tumor cells activates the P2X4 receptor on neighboring cells, leading to intracellular mTOR activation, which, in turn, induces reactive oxygen species (ROS) production, thereby promoting tumor progression. The authors further showed that the combined administration of chemotherapeutic agents with P2X4 inhibitors leads to an extensive tumor regression in colon carcinoma preclinical models [95]. On the other hand Zhou and colleagues attributed a role to P2X4 in antitumoral immune reactions by identifying its role on M1 tumor infiltrating macrophages [96]. These authors showed that incubation of cancer cells with M1 macrophages reduced CRC growth in vivo and that this phenomenon was dependent upon expression of P2X4 on M1 and activation of CD8^+^ lymphocytes. They also demonstrated a reduced expression of P2X4 in CRC human samples as compared to controlateral non cancerous controls [96]. This apparently contradictory role of the P2X4 receptor when expressed by tumor cells, as opposed to its role when expressed by immune cells, is only seemingly paradoxical and is also characteristic of P2X7-mediated responses [10]. Therefore, when designing antitumor therapeutic strategies targeting one of these receptors, it is essential to take into account their functions across multiple distinct cell types, potentially adopting a personalized therapeutic approach tailored to the differential levels of receptor expression in the various cellular subtypes, as determined by biopsy.

## Conclusion

P2X receptors represent important modulators of intestinal inflammation and disease progression in the colon, acting across multiple cellular compartments, including epithelial, neuronal, and immune cells. P2X1 and P2X2 receptors modulate inflammatory progression by influencing neutrophil recruitment, vascular integrity, and enteric glial activation, thereby affecting mucosal barrier function and neuroimmune homeostasis. P2X3 and P2X4 receptors play a prominent role in neuronal sensitization and visceral pain, while P2X4 and P2X7 regulate epithelial and immune cell responses by controlling cytokine release, inflammasome activation, and immune cell survival. Notably, P2X7 emerges as a key regulator of immune balance in colitis, shaping T-cell polarization, macrophage function, and fibrotic remodeling. Within the tumor microenvironment of colorectal cancer, where extracellular ATP levels are persistently elevated, P2X receptors, particularly P2X7 and P2X4, act as potent modulators of tumor cell behavior. Their activation promotes tumor growth, angiogenesis, metabolic reprogramming, therapy resistance, and metastatic dissemination through signaling pathways such as NF-κB, PI3K/AKT/mTOR, and through the regulation of extracellular vesicle release. At the same time, P2X receptor expression on tumor-infiltrating immune cells can support antitumor immunity. Overall, the functional outcomes of P2X receptor signalling are highly dependent on cellular context, with distinct and sometimes opposing effects arising from receptor expression in tumor versus immune cells. These features highlight the need for therapeutic strategies that account for receptor distribution and disease stage. Overall, a deeper understanding of P2X receptor biology in the colon may facilitate the development of more selective and personalized purinergic-based interventions for both inflammatory and neoplastic intestinal diseases.

## Data Availability

No datasets were generated or analysed during the current study.
